# Community pharmacist involvement in social prescribing for mental health: a qualitative study

**DOI:** 10.1017/S1463423624000409

**Published:** 2024-12-20

**Authors:** Denise A. Taylor, Andrea D.J. Taylor, Matthew Jones, Hannah E. Family

**Affiliations:** Department of Pharmacy and Pharmacology, University of Bath, Claverton Down, Bath and Northeast Somerset, UK

**Keywords:** Community pharmacy, community pharmacists, general practitioners, primary care, social prescribing

## Abstract

**Aim:**

We aimed to explore participant perspectives on social prescribing (SP) for mental health and well-being and the acceptability of community pharmacists (CP) as members of SP pathways that support people with mild to moderate depression and anxiety.

**Background:**

SP aims to support people with poor health related to socio-demographic determinants. Positive effects of SP on self-belief, mood, well-being, and health are well documented, including a return to work for long-term unemployed.

**Methods:**

The study was set in a city in southwest England with diverse cultural and socio-demographics. We recruited SP stakeholders, including CP, to either one of 17 interviews or a focus group with nine members of the public.

**Findings:**

An inductive iterative approach to thematic analysis produced four superordinate themes: (1) offering choice a non-pharmacological option, (2) supporting pharmacy communities – ‘it is an extension of what we do’, (3) stakeholder perspectives – pharmacists are very busy and their expertise unknown by some, and (4) potential for pharmacy in primary care.

Stakeholders viewed CP as local to and accessible by their community. Pharmacists perceived referral to SP services as part of their current role. General practitioner participants considered pharmacy involvement could reduce their workload and expand the primary healthcare team. Importantly, general practitioners and CP viewed SP as a non-pharmacological alternative to prescribing unnecessary antidepressants and reduce associated adverse effects. All participants voiced concerns about pharmacy dispensing busyness as a potential barrier to involvement and pharmacists requesting mental health training updates.

Key findings suggest CP offer a potential alternative to the general practitioner for people with mild to moderate depression and anxiety seeking access to support and health information. However, CP need appropriately commissioned and funded involvement in SP, including backfill for ongoing dispensing, medicines optimization, and mental health first aid training.

## Introduction and background

Social prescribing (SP) connects people to social activities to promote social engagement and self-care and improve self-confidence and self-actualization (Eaton, 2020). These social activities are all locally based to connect people to their local community in primary care. Such activities support people on a psycho-social level, allowing them to develop skills, meet people, and engage in communication or joint activities (such as singing or art), which can improve mental health and well-being (Cohen and Wills, 1985; Uchino *et al.*, 2012; Drinkwater *et al.*, 2019). Because these social activities are prescribed, the individual incurs no cost as these are funded by local commissioning or charity groups (Eaton, 2020). However, the venue may have transport costs, so locally embedded pathways were preferred so people could walk to them. SP also contributes to the concomitant removal or reduction of social stressors (Cohen & Wills, 1985; Uchino *et al.*, 2012), which supports improving mental health indices (Cohen and Wills, 1985; Uchino *et al.*, 2012; Drinkwater *et al.*, 2019). This is achieved by patient advocates or the Link worker in the SP pathway directly contacting advisory services, including social, housing, and or benefits agencies, to facilitate access to available and appropriate services (Grant *et al.*, 2000; Andermann and CLEAR Collaboration, 2016; Webber & Fendt-Newlin, 2017).

Taking a social determinant of health approach enables the person to be seen as an individual rather than a patient or a cultural bias (Andermann and CLEAR Collaboration, 2016). Importantly, in October 2018, Theresa May, the then Prime Minister of England, announced that SP was the first-line treatment for people with low mood and anxiety stemming from loneliness in England (May, 2023), and ‘Tackling Loneliness’ the formal policy document was published in June 2021 (Macdonald *et al.*, 2021). Importantly, this policy endorsed the need for providers of social activity programmes to work with local General Practice organizations within primary care settings to ensure that referrals to local health practitioners were available for users of social activities to enable ready access as needed for healthcare advice and support. Indeed, the Community Wellbeing Service model developed by Gloucestershire enabled social activities link worker to be based in each General Practice. Hence, referrals bypassed the general practitioner and were made directly by the nurse practitioner or receptionist. Such schemes enabled the general practitioner to support urgent, complex patients (Gloucestershire County Council, 2023).

The value of SP in mental health is becoming more evident as social determinants (Andermann and CLEAR Collaboration, 2016) influencing both physical and mental health, including housing, income, and education, are increasingly recognized (Allen *et al.*, 2013; Council on Community Pediatrics, 2016).

Importantly, the link between the lack of or harmful social support and its resultant stress on the individual has been linked to negative impacts on physical and mental health, including increased risk of cardiovascular disease (Uchino *et al.*, 2012).

A randomized controlled trial concluded that providing or prescribing social activities increased an individual’s perceived overall health and reduced anxiety (Grant *et al.*, 2000). Furthermore, a systematic review concluded that social participation interventions that supported people with mental illness to develop community networks enabled them to establish wider social networks to improve mental health outcomes (Webber& Fendt-Newlin, 2017).

A further study analysed the dialogue on the need for antidepressants or talking therapy between 52 general practitioners (GPs) and their patients (Ford *et al.*, 2019). Most patients did not want pharmacological therapy, although they ended up with this treatment. The authors concluded that currently available treatments, such as antidepressants or talking therapy, do not meet the needs or wants of the patient and that SP offers a valuablechoice. It is important to note that there is little evidence to suggest that antidepressants are effective in mild or even moderate depression. However, they do place the recipient at risk of adverse effects, including suicidality, bleeding, and increasing anxiety (NICE Guidance 222, 2022).

The Royal Pharmaceutical Society of England is actively promoting the clinical and communication expertise of community pharmacy practice pharmacists and their understanding of when medicines are beneficial or not as a critical component for pharmacists to be involved in SP pathways (Royal Pharmaceutical Society of England, 2023) Furthermore, the Oxford SP network (Akinyemi, 2021) has highlighted that pharmacists need to become more involved with SP by actively referring customers identified as having social needs related to loneliness or other factors associated with the aftermath of the COVID-19 pandemic.

CP possess high levels of interpersonal and communication skills (Akinyemi, 2021; Royal Pharmaceutical Society of England, 2023) and are trained experts at diagnosing and managing minor ailments. They also safely and legally dispense medication, deliver local public health initiatives to their local community members, and develop strong relationships with patients. A crucial part of their clinical services focuses on supporting people with long-term conditions, including mental health, and, or referring patients to members of the primary healthcare team and secondary services as appropriate for the individual. No appointment is required to see a pharmacist, and people attend when they are well and when they are unwell to receive medication and health advice. This means pharmacy team members are well placed to identify people who may benefit from SP and refer them appropriately to local services or SP activities. Community pharmacies are also generally open longer than medical surgeries, with some open 100 hours each week, meaning weekend and after-work consultations with a pharmacist are available without an appointment. These organizational characteristics could facilitate SP to be physically accessible to more people more quickly.

Level One Healthy Living Pharmacies (HLP) (Brown *et al.*, 2014) offer a broad range of health promotion interventions through community pharmacies designed to meet local needs, improve the health and well-being of the local population, and help to reduce health inequalities (Brown *et al.*, 2014). A systematic review demonstrated that brief interventions delivered in the community and primary care were inexpensive and cost effective (Wilson and Suhrcke 2016). HLPs might provide an ideal environment for referral to and/or provision of SP services as all staff are trained to support mental health and well-being (Brown *et al.*, 2014). In 2018, one study reported the perspectives of pharmacists and pharmacy technicians towards SP (Taylor *et al.*, 2019). Of the 120 participants, 86 respondents (89.6%) believed pharmacists should be involved (Taylor *et al.*, 2019). To date, no research has explored SP stakeholders, members of the public, or perspectives of CP’ involvement in SP.

## Aims

This study aimed to explore public, SP stakeholders, and CP’ perspectives on the acceptability of CP as members of SP pathways that support people with mild to moderate depression and anxiety. This paper addresses three research questions:

1. What are public, community pharmacist and SP stakeholders’ perspectives on SP for mental health and wellbeing?
2. What are public and SP stakeholders’ perspectives on community pharmacy inclusion in a mental health SP pathway?
3. What are community pharmacist perspectives of involvement in a mental health SP pathway?

## Methodology

We took a pragmatic exploratory approach to our study design as there was no precedent in this research area (Kaushik and Walsh, 2019). This enabled us to use multiple methods, including thematic analysis as per Braun and Clarke (Braun and Clark, 2006), and recruit participants who wanted to share their experiences and perspectives. Pragmatism embraces each person’s experience as a unique experience that may or may not be similar to others (Kaushik and Walsh, 2019). For this reason, we did not aim for data saturation in data collection due to the anticipated rich diversity of our participants’ experiences (Braun and Clark, 2006). We also did not adopt a theoretical framework as we were generating new knowledge in an area where poor socio-demographics have been linked to poor mental health. We did not want our findings to be potentially restricted by such a framework in the analytical process (Kaushik and Walsh, 2019). However, we did aim for inductive thematic saturation (Braun and Clark, 2006). This exploratory qualitative study was conducted between November 2016 and January 2017 in a city in southwest England. Data were collected using semi-structured interviews and focus group to enable flexibility of time and place for potential participants. An inductive and iterative thematic approach to analysis and reflexivity was used to ensure preconceptions on SP were challenged appropriately. Our reflexive approach included DAT, ADJT, and HEF reading and coding all transcripts and meeting to agree themes and develop a shared understanding of the findings. This occurred over a series of five meetings, with MJ then commenting on the robustness of coding until we reached an agreement.

The study was supported by a Project Advisory Board (PAB), including national and local SP experts, CP, health service commissioners, and a Patient and Public Involvement Group. The latter group contributed to the study design by reviewing study information for appropriateness and the recruitment processes for public participation.

## Participants and recruitment

Inclusion criteria for recruitment of potential participants were adult members of the community who spoke English and representatives of key participant stakeholder groups: CP, GPs, and other SP stakeholders (including commissioners, third sector organizations, and SP coordinators (or link workers) and members of the public. Between 5 and 10 participants from each stakeholder group were sought due to budgetary and time considerations. All participants received a gift voucher as a token of appreciation for their time and contribution to the study. Refreshments were provided at the focus group as a way of breaking the ice and enabling people to relax and feel able to share their stories.

Members of the public were recruited through flyers and emails to local mental health charities and existing SP services in the area. To be eligible, participants needed to be over 18 and fluent in English, as we had no funding for translators. No prior experience or knowledge of SP or anxiety and depression was required. GPs and CP were recruited through mailed invitations to their practice or pharmacy manager, which contained a study invitation letter, information sheet, and expression of interest form for distribution to potential participants. This mailing was followed up by phone calls to the practice or pharmacy manager to provide further information about the study and invite expressions of interest.

SP stakeholders were recruited through snowballing after identification by members of the PAB and study participants as having essential local knowledge of SP. Due to the nature of our recruitment process, it is unknown how many people did not take part in our study, but after expressing an interest in taking part, there were no dropouts.

## Data collection

At recruitment, participants participated in a single one-to-one interview (either face-to-face or by telephone) or a focus group. All participants received a study information pack and a summary of the topic guide ahead of the interview/focus group date.

**Figure 1.**
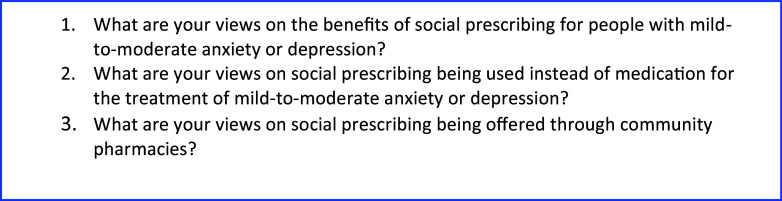
Summary of topic guide questions.

One semi-structured topic guide was developed for interviews with GPs, CP, and stakeholders, with a separate topic guide for interviews and a focus group with members of the public. These were reviewed by a member of the public and a GP. Following this peer review, the topic guides for the GPs, CPs, and stakeholders were piloted in a focus group and interview setting before data collection commenced. Figure 1 summarizes the topics covered in the interviews and focus group.

Before the interview or focus group started, participants provided written consent (for telephone interviews, this was returned to the study team before the interview and confirmed verbally during the interview). Interviews lasted between 26 and 63 minutes and the focus group 48 minutes. The interviews and focus group were audio recorded using a digital recorder and transcribed verbatim, with field notes taken as appropriate. Participants were thanked and debriefed at the end of the interview or focus group.

## The research team

At the time of the study, DAT (PhD, registered pharmacist), ADJT (PhD), and HEF (PhD, registered psychologist) were experienced qualitative researchers, and MDJ (PhD, registered pharmacist) joined to refine qualitative skills. DAT was the lead researcher and was responsible for analysis and delivering project objectives. All were responsible for participant recruitment and data collection, held academic research positions, and knew little about SP, but were interested in professional and public perceptions of it as an alternative therapy to medication. Preliminary meetings with SP stakeholders enabled us to establish potential participant networks and ascertain interest in the study area. DAT completed two telephone interviews and facilitated the focus group with ADJT. ADJT and HEF completed seven in-person interviews each, and ADJT and MDJ conducted one in-person interview.

## Data analyses

Thematic analysis was chosen as the method for data analysis due to its flexibility because it is not tied to any specific epistemological position. An iterative and inductive thematic analysis was completed using the six steps outlined by Braun and Clarke (2006) (1) familiarization with the data; (2) generation of initial codes and refining of codes; (3) searching for themes which were then (4) named and defined; (5) themes, names and definitions were reviewed before proceeding and to (6) writing of the report.

## Ethics

Before commencing, the study was reviewed and approved by the Research Ethics Approval Committee for Health (REACH) at the University of Bath (reference number EP/16/17 069). Health Research Authority approval to conduct research with National Health Service staff was given on October 26 2016 (reference number: IRAS 214818).

## Results

### Participants

Twenty-six participants were recruited, with 17 interviews (six CP, six GPs, four SP stakeholders, and one member of the public) plus one focus group with nine members of the public being held at a local health activity centre. Ten interviews were face to face at a workplace or mutually agreed upon setting, and seven were by telephone. Due to time restrictions and a limited study period, it was not feasible for participants to comment on their transcripts. Table 1 depicts the study participant demographics. The participant identifier is used as a pseudonym in all quotations.

**Table 1.** Participant demographics

Participant	Group	Gender
CP1	Community Pharmacist	F
CP2	Community Pharmacist	F
CP3	Community Pharmacist	M
CP4	Community Pharmacist	F
CP5	Community Pharmacist	M
CP6	Community Pharmacist	M
GP1	General Practitioner	M
GP2	General Practitioner	M
GP3	General Practitioner	M
GP4	General Practitioner	F
GP5	General Practitioner	M
GP6	General Practitioner	F
SPS1	National Expert; SP Commissioner	F
SPS2	National Expert; Charity Provider	F
SPS3	SP Coordinator	F
SPS4	SP Coordinator	F
MP1	Interviewee	F
MP 2-10	Focus Group Participants (FGP)	Mixed

### Analysis

Three team members (DAT, ADJT, and HEF) processed all data, codes, and themes, agreeing on each stage before progressing. Themes were coded manually in Microsoft Word due to researcher preference. An inductive iterative approach to thematic analysis resulted in three superordinate themes: (1) Offering Choice – a non-pharmacological option, (2) Community Pharmacist Perspectives of Prescribing Social Activities for Mental Health, and (3) Pharmacy Practice – unknown territory for some GPs. The thematic hierarchy is outlined in Table 2, and the results are presented here.

**Table 2.** Thematic hierarchy

Number	Title
1	Offering Choice – A Non-Pharmacological Option
1.1	Social Prescribing for Modern Life
1.2	The Burden of Mental Distress and Deprivation
1.3	Moving Away from Antidepressants – Current Practice not Evidence-Based
1.3.1	Social Prescribing Offers Patient Choice and Informed Consent
2	Supporting Pharmacy Communities – ‘It’s an Extension of What We Do’
2.1	‘It’s an Extension of What We Do’
2.2	Community Pharmacist Perspectives of Prescribing Social Activities for Mental Health
2.2.1	Identifying Potential People for Referral
2.3	Potential Enablers and Barriers for Pharmacy and Social Prescribing
2.3.1	Enabling Perspectives
2.3.2	Community Pharmacist Perspectives – Wanting to be Involved but Knowing the Reality
3	Stakeholder Perspectives- Pharmacists are Very Busy and Their Expertise Unknown by Some.
3.1	Too Busy to be Involved?
3.2	Pharmacy Practice – Unknown Territory for Some General Practitioners
3.3	Pharmacy Opportunities in Social Prescribing and Primary Healthcare
3.3.1	Preferred Community Pharmacy Partners for Social Prescribing
3.4	Pharmacy Opportunities in Primary Healthcare
3.4.1	Community Pharmacy and Pharmacists – An Underused Resource

## Offering choice – a non-pharmacological option

In this theme, GPs shared their concerns on how the breakdown of the extended family has left young people without access to the wisdom of aunts, uncles, and grandparents, whom the young have historically turned to for advice instead of their parents. GP participants now had more young people seeking appointments to discuss their concerns about surviving day-to-day stressors. There was also increasing recognition that because there were seemingly appropriate medicines available, life events were being medicalized rather than being managed as life events.

### Social prescribing for modern life

*‘Where I work. we are seeing a burden of mental health problems and the mental health problems in young people seem to be due to a sort of lack of general resilience and ability to live on your own. I don’t know what’s going on, but something is making them all very vulnerable and unable to cope so any of these sort of schemes (SP) where they are actually being encouraged to socialise and to access life skills which will help them’.* (GP4)

*‘A lot of people’s problems have been existing for centuries before drugs were available to deal with them, and it’s only recently that we think that the answer to people’s loneliness or low mood is a selective serotonin reuptake inhibitor’.* (GP1)

#### Medicalizing life events

*‘You know maybe we shouldn’t really be medicalising it in any way shape or form mild anxiety and depression, we should just be giving positive encouragement and that is about it really. Otherwise, we have medicalised it you know? We are in a time now where life experiences become illnesses but they are actually life experiences and you know there aren’t necessarily medical solutions to them at all’.* (GP2)

### The burden of mental distress and deprivation

GPs shared how at least 20% of their patients were lonely, depressed, stressed, or distressed by their current life circumstances of deprivation. Our SP expert shared that 48% of people attending SP pathways were also troubled by low mood or poor mental health and well-being.

*‘Yeah so there is a lot of you know lonely depressed and stress, distressed patients we have to deal with in our surgeries. I think it would probably make up a substantial portion I would probably say 20% at least’.* (GP5)

*‘So referrers (of people to SP) were asked to identify a reason for referral 48% mentioned something about low mood or anxiety or mental health and wellbeing’.* (Expert 1)

## Deprivation

*‘The demographics are deprived and very deprived patients who have a lot of single-parent families, we have quite a lot of elderly patients in the area as well who live alone, and we have quite a lot of issues with unemployment, poverty or financial difficulties, relationship difficulties and substance misuse. Yes I think we are probably eighth in the most deprived practices in [city]’.* (GP5)

### Moving away from antidepressants

Healthcare professionals shared their frustration at being limited to prescribing antidepressants for people who did not need medication but needed social support to develop strategies to survive modern life. One GP shared that in the future, the current practice of prescribing antidepressants first line would be viewed as unethical, as prescribers did not give sufficient information on treatment options for patients to make informed choices.

*‘I am fed up with the fact that we all seem to be prescribing antidepressants left, right and centre. I am not sure it is the way to go. I think we need to be doing lots of other things. There are plenty of other ways to help people feel better, and I think these sorts of schemes* (SP) *would be great, especially if they are run by people in the voluntary sector because it is a win, win situation then’.* (GP4)

*‘I would say that 10 years from now we would look back on what we are doing now, and we would consider it unethical, because for instance, if I am going to advise you to take a medication ok, and I don’t advise you the numbers needed to treat and the numbers needed to harm, you have to say how can that be ethical? I haven’t really given you the information for you the patient to make a proper choice’.* (GP2)

#### Social prescribing offers patient choice and informed consent

In this section, CP, GPs, and social prescribers comment on the inappropriateness of prescribing antidepressants for people with social determinants of poor health and well-being. In particular, this discussion highlights the need for informed consent for any treatment, which includes evidence for successful and potentially harmful outcomes. Pharmacist patients often wanted their medication list to be reduced rather than expanded. Consequently, pharmacists viewed SP as an effective non-pharmacological option, reducing medication burden and potential patient harm.

*‘Umm I think they should be able to choose with informed consent so I think if we are giving them enough information to be able to explain all the benefits and risks of both options I think patients are quite likely to pick social prescribing as a first option the same as if someone is diabetic they want to try with diet and exercise before they are given any tablets’.* (CP2)

*‘I’m not a clinician, but I know that people can have their anxiety and depression managed in lots of different ways, CBT and medication and then exercise also increases serotonin so that it makes people feel better’.* (SPC1)

*‘A lot of med reviews that I do, people would like say ‘oh I hate taking tablets’ or ‘I don’t want to take any more tablets what can I do?’ So that would have quite a big impact on my patients, particularly, I think to be given out their prescription saying you could also do this which might help which might mean we could reduce the medication in future’.* (CP2)

#### Patients and Doctors want a ‘quick fix’ solution

Our general practitioner participants said they believed patients and doctors wanted a *‘quick fix’* to the problem shared in the consultation room. For doctors, a prescription was quick and easy to write, and they perceived this as giving the patient what they wanted: a quick-fix solution to their problem. A medication released the patient from having to actively engage with their condition with behaviour change therapy, such as losing weight or exercising more, as the patient perceived these as being too difficult or taking too long. Doctors viewed this culture of *a ‘quick fix’* for their problem as a potential barrier to engaging in SP activities.

*‘I think one of the biggest barriers, which I’m not sure you’ll pick up on, one of the biggest barriers to this (SP) at the moment, is our culture of wanting, both in terms of GPs wanting to give a quick fix and patients wanting a quick fix…. they want to come away with a prescription in their hand. Something that says, ‘I’ve got the answer to this’”.* (GP1)

## Community pharmacist perspectives on prescribing social activities for mental health

In this theme, CP shared their perspectives on social activities for mental ill health but also their concerns on the use of antidepressants first line for people with low mood as a consequence of life events. Their day job involved talking to people in such scenarios and offering advice on medication and non-pharmacological supportive approaches to improve mental health and wellbeing.

We asked CP if they wanted to refer people to SP activities to support well-being and good physical health. Maybe unsurprisingly, they were already involved in referring people to social activities, including Slimming World, exercise, and smoking cessation. As part of their role, the community pharmacist would follow-up the progress and satisfaction of their client and their perceived benefits. As Community Pharmacist 5 suggests, referral to exercise or activity groups was something that they were already involved with.

*‘It’s an extension of what we do. They* [the socially prescribed exercise or activities*] have been brilliant because what we do is we like to follow the patients up as well, so for example, if we were to refer someone to Slimming World [registered weight loss programme] we would ask them to come back in 4 weeks’ time, weigh them, talk to them see how they are getting on and they find it really helpful and because it has been free for them and they have come to see me in the first place they are in the right frame of mind and they are losing weight so I mean we have had people come back and have lost a lot of weight and that in turn has improved their health’.* (CP5)

### The potential role of the community pharmacist in social prescribing activities

This sub-theme explores the place of community pharmacy practice within SP from the perspective of CPs, GPs, the public, and SP stakeholders. It highlights CP participant perspectives separately from other stakeholders to explore contrasting viewpoints. CPs perceived that SP could become part of their daily role of signposting and giving healthy living advice to community members and believed supporting SP would benefit the pharmacy profession. However, they worried about having the time to be fully involved. As well as dispensing prescriptions, CPs provide advice, information, and local public health initiatives to their customers and patients, including counselling on minor ailments and signposting to other services. Many provided advanced clinical services, including blood pressure monitoring and vaccinations.

*‘I think most community pharmacies are, they carry information about local services so there is always posters and leaflets and people do go into the pharmacies and expect to see local healthcare information…I would say this* (SP) *would be something that would, should be standard provision for pharmacists at the moment, so it would only be an extension of what we do already’*. (CP1)

Some CP participants were a HLP, and others were not. However, all perceived community pharmacies as beneficial for SP because of their extended opening hours and the potential for their time to be restructured to provide space for SP referrals in less busy periods.

*‘I always think that the advantage of the community pharmacies that they are open to everyone open, usually very long hours and with no appointment,* (this) *is also very often the downfall as well because it can be quite difficult to regulate how you do divide up your time’.* (CP1)

CP believed they offered a safe space, had confidential consultation rooms, were trusted healthcare professionals, and supported people who did not see their GP, such as the homeless, young people, and those without chronic medical conditions. Their role in New Medicines Service (NMS) was seen as a possible way of identifying people appropriate for SP.

*‘Pharmacies are ideally placed because we are seen as quite a safe space… we are seen as safe; certainly, pharmacies are seen as trusted professionals. We also have a very privileged opportunity to see people and to maybe identify people who have mild to moderate depression’.* (CP5)

*‘… but a lot of people that use pharmacy do not see a GP, so yeah, so we can pick up those people that haven’t been to the doctors in a long time. We recognise that they need some sort of help or referral or something’.* (CP6)

#### Identifying potential people for social prescribing referral

CP participants could see the potential benefits of being involved in an SP pathway, as it supported CP to be seen as a place of health advice embedded within local primary health service pathways. Importantly, CPs felt their current consultation style, which encompassed identifying patient ideas and concerns, as well as their agenda, and goals, was a good grounding for SP consultations.

*‘We kind of link that* (SP) *into what the other services that we provide so we’re speaking to some of those patients; maybe part of the MUR* (Medicines Use Review) *or NMS or just any other normal consultation…just one of the things that we will provide* (SP) *because we are aware of it, we’re able to say ‘oh this is a facility that is very close to us and this may be something that you could benefit from’”.* (CP3)

*‘We get involved in health promotions and public health campaigns. If it was something like that where we were given sufficient information about a scheme and how you would signpost someone onto it, and what the scheme involved, it would be something that could quite easily be initiated in community pharmacy if there is sufficient information about how it was being run and who was in charge’.* (CP1)

‘*It promotes the idea of a community pharmacy being part of the healthcare team as a whole as opposed to just being retail outlets’.* (CP1)

‘(As a pharmacist) *you know the majority of your patients. You see them on a daily basis, you see them with the same drugs whether it be for low-mood drugs, a number of things,… I had a patient who had just gone through a divorce. Quite a difficult divorce umm and she felt she couldn’t talk to anyone umm and she was really down umm her husband had cheated on her and it was quite traumatic so umm she had been put on an antidepressant umm a low dose of citalopram to help her through. In my opinion, I don’t think that was quite necessary. I think if she had had a social network or social support group to help her or if I had been equipped with the right skills to help her we may have not had to go down that route’.* (CP4)

Some pharmacist participants felt they would require updating in signs, symptoms, and condition-specific information on depression and anxiety, which could be provided via internet discussion groups and formal continuing professional development such as completing the Mental Health First Aid programme (Anon, Mental Health First Aid, (MHFA). The MHFA certificate teaches pharmacological and non-pharmacological options for treating depression and anxiety and supporting patients. GP participants supported this potential option for non-pharmacological approaches to mental health and suggested conversations pharmacists could have with their patients.

*‘There is no particular reason why they couldn’t also have a role in, umm you know, determining what the patients actually want to achieve and actually saying ‘well here is another way of doing it, a non-drug way of doing it’, so there is, and they would probably have a lot more time’.* (GP2)

*‘I think that could work quite well, that they give out, that’s an intelligent idea, they give out their antidepressants and saying ‘have you heard of this service’ … I guess it’s harder for them to do in an in-depth consultation of what somebody might need, that’s the only thing’.* (GP6)

### Potential enablers and barriers for pharmacy and social prescribing

#### Enabling perspectives

Enablers of pharmacy involvement for SP in mental health included educating the general public about pharmacists, their expertise in medicines, health and well-being, and the availability of private consultation rooms. There was also an increasing necessity for good electronic communication links with local GPs, including access to GP electronic prescribing and patient record systems to facilitate urgent referrals. GPs believed they could work collaboratively to support distressed patients.

*‘Again, I think this is coming down to changing the culture and the ethos, and if you’re going to do that, then people need to know that they can go to their pharmacist without necessarily going to see a GP for a problem relating to low mood or anxiety, and that is a great thing’.* (GP2)

‘*We could use pharmacy much, much more. Pharmacists, I would have no objection to them having an EMIS (an electronic GP record system), having use of the computer online there so they can just look up and see what the GP said and look at umm, it would be read-only and audit trail, but then they could see more patients and if they think somebody needs to be seen I would be quite happy for them to book an appointment for somebody’.* (GP3)

*‘I think patients would need educating that they could talk to us in private. I think if they knew they could have use of the consultation room no matter what it was, and could talk to us confidentially. But…I have had people just all of a sudden pouring their heart out to you over the counter, regardless of how many people are behind them, which I think is alarming because it either shows just how much they want some help and they just want to talk to anyone or that some patients are quite happy just to talk freely’.* (CP2)

#### Potential barriers for pharmacy and social prescribing

CP were under pressure with heavy workloads, financial constraints, and reduced staff numbers, with many CPs working in isolation, combining to increase the pressure of day-to-day work.

*‘I think the very real risk is that the practicalities of actually doing that are very, very difficult just because of the workload in pharmacies that, I think, probably every single community pharmacist that I could think of would say that they never have enough time to spend with the customers as it is, and I’m sure GPs would feel pretty much the same. To then add it to every consultation, additional questions about their general wellbeing that might flag them up as someone that could benefit from something like this, just think the time constraints is quite large’.* (CP1)

#### Stakeholder perspectives – pharmacists are already too busy

Stakeholder participants shared various concerns about CPs engagement with SP, mainly centring on the busyness of the pharmacy in terms of dispensing commitments.

The general consensus from focus group and GP participants was that CP were very busy. They would need to schedule SP referrals to less busy times of the day and or have impromptu interactions with patients.

*‘I don’t know what other people’s pharmacies are like but the one that I go to is absolutely heaving, and it is a very busy place so possibly not the best place to be having that sort of conversation* (about low mood)’. (FGP)

*‘I think it would be two-way, and obviously, when they are in the heat of battle when it is really busy, they ain’t going to do it. But if it is a bit quieter, they can say you know “you look a bit fed up; how is it going?” You know, ‘have you heard about this?’ and yes, promote it’.* (GP3)

## Pharmacy practice – unknown territory for some general practitioners

### Unfamiliarity of pharmacy skills and expertise

Although some GPs talked about expanding the primary care health team by including community pharmacy involvement in SP, some GPs had little understanding of the role of CP. For example, their expertise with medicines, diagnosing and treating minor ailments, and their ability to talk to and counsel people on medication or medical conditions, including mental health.

The lack of knowledge and understanding of CP’ training, role, and scope of clinical and medication knowledge that some GP participants held was a barrier in itself to the potential involvement of CPs in SP referral. For example, some GP participants felt that only a GP was likely to discover that a person had a mental health problem within their consultation, not the pharmacist. This belief was based on their supposition that a patient would not want to talk to their pharmacist about mental illness.

Another GP participant was unclear about the actual role, training, or capability of CPs. GP1 had little understanding of pharmacists’ expertise in medicines use and whether they should be checking the prescription with the patient. As medicine experts, pharmacists always check the prescription with the patient to check if these are the medicines they were expecting. Anecdotal evidence suggests that there are errors on one in five prescriptions, so this is an important safety check for pharmacists.

*‘Is it ok for the pharmacist to say, ‘I notice that you’ve been issued this medication, are you aware that there are also these things available to you that might be helpful for this particular problem?’ Well is that ok? Well, I don’t know, because are pharmacists supposed to comment on the reason why people are being issued the medication that they’re given? I don’t know, that might be a problem’.* (GP1)

*‘It is interesting, isn’t it, because you started off by saying mild to moderate anxiety and depression and I am thinking it is not the sort of thing that people go to their pharmacists to discuss…They are much more likely to go to the pharmacist about their eye complaint or their sore throat or their cough or whatever, so it’s that opportunity’.* (GP4)

### Pharmacy opportunities in social prescribing and primary healthcare

This closing section outlines one general practitioner’s concerns about the variation between different pharmacy organizations and how they operate while identifying preferred pharmacy organizations to be involved with SP. Importantly, another GP recognized community pharmacy practice as not being paid for what it actually does, and this limits their potential involvement in wider projects in primary healthcare.

‘*The smaller independent pharmacies and they are the ones that I have often seen doing a really good job and they have built their little consulting room and stuff. They may well take it on* (SP), *but again, they are not being rewarded properly for what they do, and they are up against the big multinationals* (large pharmacy organisations), *so I think it is a very complicated area really’.* (GP4)

GP and other stakeholders believed community pharmacy practice could take on more significant roles in the community and primary care, as some participants perceived CPs as being underused and under-recognized for their expertise. Importantly, some GP participants felt CP offered an alternative for patients to see a pharmacist rather than their GP. This was seen as an opportunity to reduce GPs’ workloads and effect efficiency savings in primary healthcare.

*‘We* (The GP Practice Team) *were having a discussion about this, about pharmacists, because we are trying in our practices to get patients to go to the pharmacists as a first point of call you know, for lots of things, because there is so much they can do’.* (GP4)

*‘I think there is probably a massive amount of efficiency savings to be made if people did do that because it would save a lot of money in terms of primary care and people having to see their G.Ps’.* (GP1)

*‘I think at the moment not everybody uses pharmacies to their full extent so, so minor illness schemes, medications reviews, I don’t think everybody is fully on board with what they can do. And I know that our GPs, engage with the pharmacist as much as we can for obvious reasons but if they were to take on a role around social prescribing, then I think that could only be a good thing because it would potentially relieve the pressure on the (GP) practices so that they can get on and do the stuff that they need to be doing’.* (SPC1)

## Discussion

### Summary of key findings

Our key findings suggest CP may be a viable alternative to GPs for members of the public with mild to moderate depression and, or anxiety who access health information or support. Community pharmacy teams currently engaged in referral and monitoring of their clients with signs and symptoms of reduced mental health taking part in social or physical activities enable an alternate health professional to approach for members of the community. Reducing the burden of GPs allows increased focus on more complex patients and results in cost savings for general practice and primary care. However, part of these savings could support CP in delivering such interventions.

We identified key barriers that would hinder the implementation of SP within community pharmacies if not accounted for in the design and implementation of pharmacy-led SP services. These included the current high level of pharmacy business and the need for pharmacist updating in the management of mental health (Anon *et al.*, 2023); both GPs and CP acknowledged that the public needed education on the skills and services pharmacists provide to reduce the need for GP consultations further. GPs further perceived CPs as being able to provide primary healthcare services cheaper than GPs, which could manifest as primary care cost savings, while also realizing community pharmacies were underfunded. GP participants highlighted the need to use their local CP’ professional skills and expertise in everyday practice to reduce their workload, have input into SP pathways, and build the infrastructure of primary care services for their patients. Importantly, similar findings have been identified in the potential for Canadian CP (Calogero and Caley, 2017) to also support SP. Similar to UK pharmacists, their funding model is focused solely on supporting dispensing prescription items. The authors state (Hussein *et al.*, 2024) that without more flexibility in funding, pharmacists would not be able to support SP pathways, resulting in the inability to decrease the workload of medical practitioners in primary care.

However, the need for sustained funding within SP teams cannot be ignored because when funding collapses, members of the public attending those social activity services become once again unsupported persons with mental health issues and at risk of more severe mental health experiences. Historically, the first SP services in the United Kingdom were funded by third-sector charities, and this precarious balance of the services failing in austere times meant job losses and health risks for participants. With funding from the National Health Service, there has been stability in the delivery of SP services. However, the funding is aimed at the link worker and not clinicians such as pharmacists (Skivington *et al.*, 2018).

Wardle (2023) highlights several methods to access funding for SP Pathways, which encompasses contacting charities, including Mind Mental Health Charity, National Lotto Charities, and government-centric funding. The amount of funding varies according to the country within the United Kingdom and associated austerity measures. A potential way forward to support pharmacists within SP may be for local GPs and pharmacists to complete joint funding bids in the future.

### Study limitations

We report findings from a small study in one city in southwest England, so findings may not reflect those of a different population sample. However, participant demographics reflected the city and provided a snapshot of the local situation. Another limitation is that most participants were passionate about SP and its benefits, and our findings may also not reflect a wider population perspective.

## Conclusions

Within the United Kingdom, SP has become the first-line option for supporting people with poor mental health and well-being mediated by loneliness and social determinants of poor mental health. The implementation of SP is set to increase in the United Kingdom as a non-pharmacological approach to improving mental health and well-being without medication-associated adverse effects. CP across England increasingly provide advanced clinical and public health services, making them ideally placed to identify people who would benefit from SP and refer them appropriately. However, our findings suggest that a robust funding model is needed to support community pharmacies to thrive within local communities. Such funding includes payment of all associated dispensing costs for medication and mental health first aid training.
